# Supplementary material for: Effective treatment of mitochondrial myopathy by nicotinamide riboside, a vitamin B3
Source: EMBO Mol Med. 2014 Apr 7;6(6):721–31. doi: 10.1002/emmm.201403943 (PMC4203351; doi:10.1002/emmm.201403943)
Supplement: Supplementary file 1 — Supplementary Figure S1 [file emmm0006-0721-sd1.pdf]

# Supplementary Information

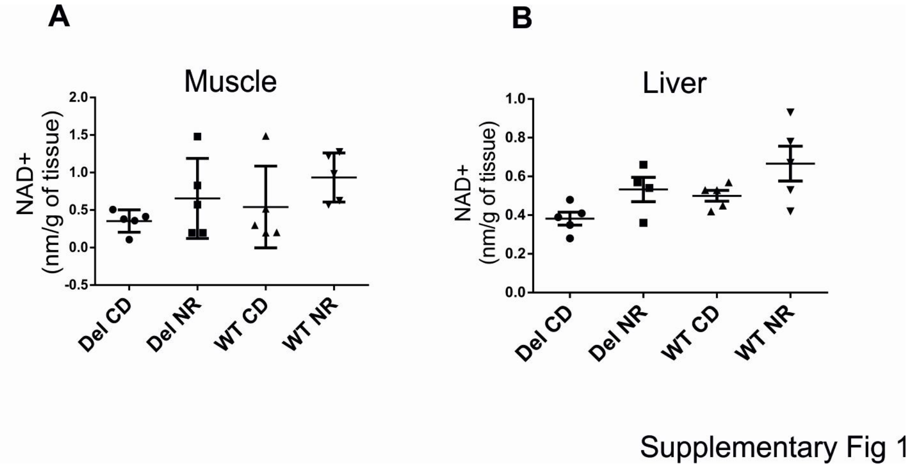


**Supplementary Figure 1. NR effects on NAD+ levels.** NAD+ levels in (A) skeletal muscle and (B) Liver in nicotinamide riboside (NR)- or chow diet (CD)-fed Deletor (Del) and wild type (WT) mice. A, ultra performance liquid chromatography and triple quadrupole mass spectrometry; B, high performance liquid chromatography.
