# Supplementary material for: Effective treatment of mitochondrial myopathy by nicotinamide riboside, a vitamin B3
Source: EMBO Mol Med. 2014 Apr 7;6(6):721–31. doi: 10.1002/emmm.201403943 (PMC4203351; doi:10.1002/emmm.201403943)
Supplement: Supplementary file 2 — Supplementary Figure S2 [file emmm0006-0721-sd2.pdf]

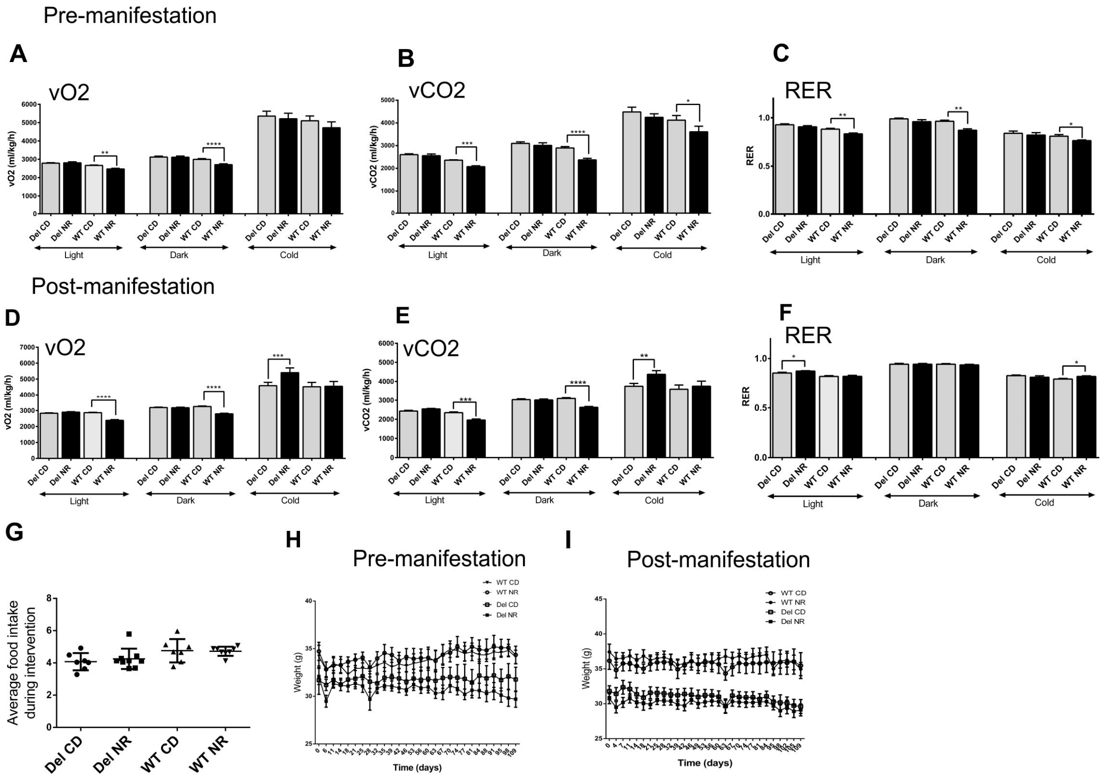

Supplementary Figure 2. Nicotinamide riboside (NR) administration induced gas exchange of Deletors upon cold exposure, and had little effects on their oxidized fuel-type in whole-organism level. CLAMS® metabolic cage analysis of (A-C) pre-manifestation (D-F) post-manifestation Deletor and their wildtype littermates after 16 weeks of NR or chow (CD) diet. Mean oxygen consumption (vO2) and carbon dioxide production (vCO2) volumes are shown, as well as respiratory exchange rate (RER), expressing the CO2 produced per O2 inhaled, indicating fuel-type consumed (1.0: major fuel source carbohydrate; 0.7 major fuel source fatty acids and ketone bodies). Values were calculated separately for mouse inactive sleeping time (light), active time (dark) and cold exposure. (G) NR did not affect food intake of any mouse group (post-manifestation). (H, I). Weight development of pre- and post-manifestation mouse groups on CD or NR.
