# Supplementary material for: Effective treatment of mitochondrial myopathy by nicotinamide riboside, a vitamin B3
Source: EMBO Mol Med. 2014 Apr 7;6(6):721–31. doi: 10.1002/emmm.201403943 (PMC4203351; doi:10.1002/emmm.201403943)
Supplement: Supplementary file 3 — Supplementary Figure S3 [file emmm0006-0721-sd3.pdf]

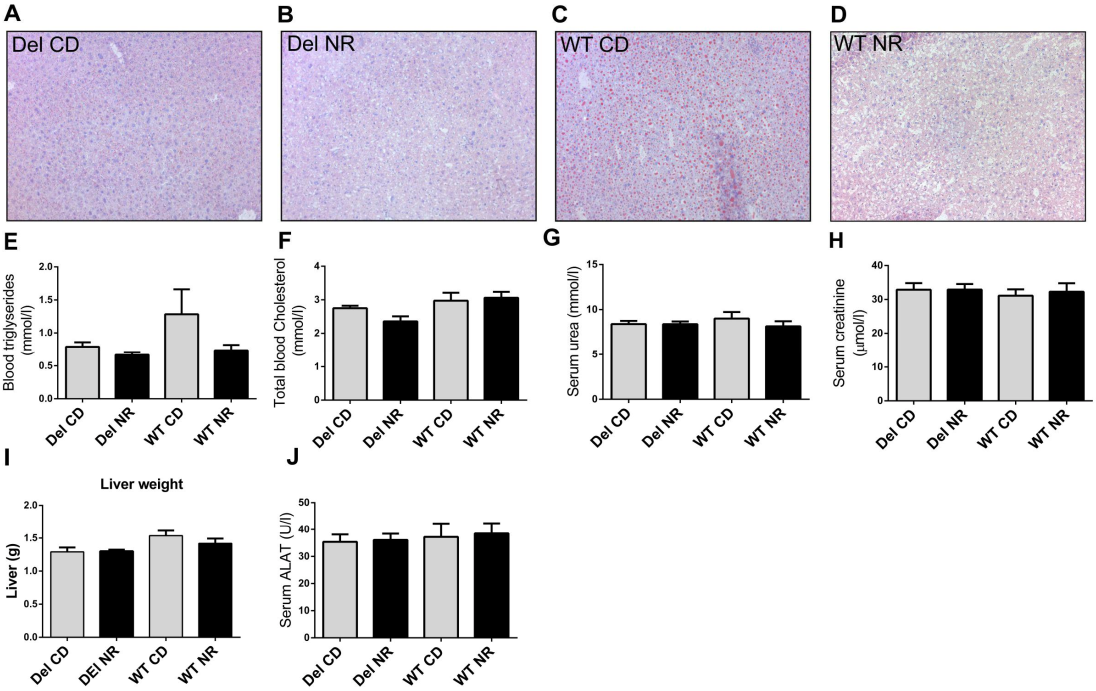


**Supplementary Figure 3. NR diet reduced liver lipid content but had no major effects on blood lipids or hepatic or renal function tests.** (A-D) Oil-red-O lipid staining with hematoxylin (blue nuclei) counterstaining on frozen sections of liver. Red droplets, most clearly seen in (C) wildtype mice on control diet (WT CD), indicate fat. (A) Deletors on control diet (DEL CD) have little fat, which is further reduced on (B) NR diet. (D)NR also reduced fat content of WT mice (WT NR). (E) Blood triglyceride and (F) total cholesterol values on NR and CD. (G) Serum urea and (H) serum creatinine levels were normal, indicating no effect of NR on kidney clearance. (I) Liver weight and (J) alanine aminotransferase (ALAT) concentration indicated no major effects on liver structure or function.
