# Supplementary material for: Effective treatment of mitochondrial myopathy by nicotinamide riboside, a vitamin B3
Source: EMBO Mol Med. 2014 Apr 7;6(6):721–31. doi: 10.1002/emmm.201403943 (PMC4203351; doi:10.1002/emmm.201403943)
Supplement: Supplementary file 4 — Supplementary Figure S4 and Supplementary Methods [file emmm0006-0721-sd4.pdf]

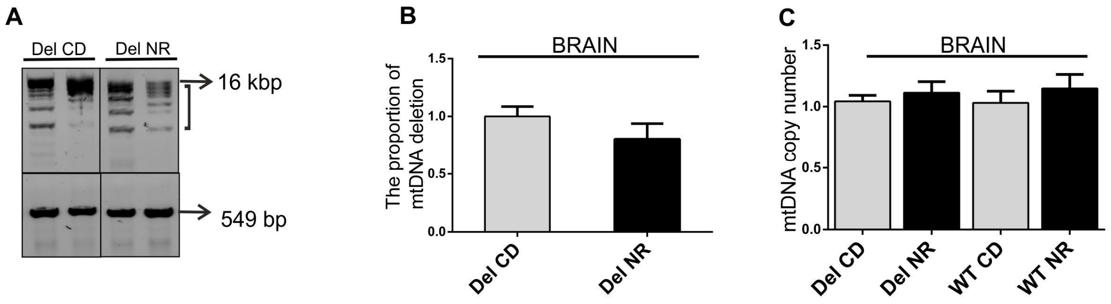


**Supplementary Figure 4.**

(A)Multiple mtDNA deletion observed in Brown adipose tissue of Deletor (B) The mtDNA deletion load in brain (C)MtDNA amount in the brain was not significantly affected by NR.

## Supplementary methods:

### Metabolomics analysis

#### *Chemicals and reagents*

All the metabolite standards, ammonium formate, ammonium acetate and ammonium hydroxide were obtained from Sigma-Aldrich (Helsinki, Finland). Formic acid (FA), 2-proponol, acetonitrile (ACN), and methanol (all HiPerSolv CHROMANORM, HPLC grade, BDH prolabo) were purchased from VWR International (Helsinki, Finland). Deionized MilliQ water up to a resistivity of 18 MΩ was purified with a purification system (Barnstead EASYpure RoDi ultrapure water purification system, Thermo scientific, Ohio, USA).

#### *Metabolite extraction protocol*

The working calibration solutions were prepared in 96-well plate by serial dilution of the stock calibration mix using Hamilton's MICROLAB® STAR line (Hamilton, Bonaduz AG, Switzerland) liquid handling robot system. Starting from a stock solution mix, 10 additional lower working solutions were prepared using water as the diluent to build the calibration curves.

#### *Muscle samples:*

Frozen muscle samples were weighed (25 – 30 mg) and transferred to Precellys homogenization tubes (Precellys 24 lysing kit, Precellys) containing 1.4 mm ceramic (Zirconium oxide) beads by adding 20 uL of labeled internal standard mix and incubated on ice for 10 min. After incubation, 30 parts of extraction solvent was added to the sample (1:30, sample:extraction solvent). In order to gain maximum recovery of small molecules, the homogenizations were performed with a Precellys 24

homogenizer (Precellys, Finland) in a two-step extraction process. In the first step, 15 parts of precooled 100% ACN + 1% FA was added to the sample and homogenized for 3 cycles of 20 sec each at 5,500 rpm with 30 sec pause between each homogenization interval. After homogenization, the sample tubes were centrifuged for 10 min at 5000 rpm at -2$^{o}$C in an Eppendorf 5404R centrifuge and the supernatant was collected in a 1.5 ml eppendorf tube. In the second step, 15 parts of 80/20% ACN/$H_2O$ + 1% FA was added to the remaining pellet and repeated the steps as above and finally pooled to the previous extract.

The collected extracts were dispensed in OstroTM 96-well plate (Waters Corporation, Milford, USA) and filtered by applying vacuum at a delta pressure of 300-400 mbar for 2.5 min on robot's vacuum station. This resulted a cleaner extract to the 96-well collection plate, which was placed under the OstroTM plate. The collection plate was sealed with the cap map and placed in auto-sampler of the LC system for the injection.

***Instrumentation and analytical conditions***

Sample analysis was performed on an ACQUITY UPLC-MS/MS system (Waters Corporation, Milford, MA, USA). The auto-sampler was set at 5°C, and the column, 2.1 × 100 mm Acquity 1.7um BEH amide HILIC column (Waters Corporation, Milford, MA, USA), temperature was maintained at 45°C. The total run time is 14.5 min including 2.5 min of equilibration step at a flow rate of 600 µL/min. Initially the gradient started with a 2.5 min isocratic step at 100% mobile phase B (ACN/ $H_2O$, 90/10 (v/v), 20 mM ammonium formate, pH at 3), and then rising to 100% mobile phase A (ACN/$H_2O$, 50/50 (v/v), ammonium formate, pH at 3) over the next 10 min and maintained for 2 min at 100% A and finally equilibrated to the initial conditions for 2.5 min. An injection volume of 5 µL of sample extract was used and two cycles of 300 µL of strong wash (methanol/isopropanol/ACN/$H_2O$, 25/25/25/25, 0.5% FA) and 900 µL of weak wash (methanol/isopropanol/ACN/$H_2O$, 25/25/25/25, 0.5% ammonium hydroxide) and in addition 2 min of seal wash (90/10, methanol/$H_2O$) were carried out. The auto-sampler was used to perform partial loop with needle overfill injections for the samples and standards.

The detection system, a Xevo® TQ-S tandem triple quadrupole mass spectrometer (Waters, Milford, MA, USA), was operated in both positive and negative polarities with a polarity switching time of 20 msec. Electro spray ionization (ESI) was chosen as the ionization mode with a capillary voltage at 0.6 KV in both polarities. The source temperature and desolvation temperature of 120°C and 650°C, respectively, were maintained constantly throughout the experiment. Declustering potential (DP) and collision energy (CE) were optimized for each compound. High pure nitrogen and argon gas were used as desolvation gas (1000 L/hr) and collision gas (0.15 ml/min), respectively. Multiple Reaction Monitoring (MRM) acquisition mode was selected for quantification of metabolites with individual span time of 0.1 sec given in their individual MRM channels. The dwell time was calculated automatically by the software based on the region of the retention time window, number of MRM functions and also depending on the number of data points required to form the peak. MassLynx 4.1 software was used for data acquisition, data handling and instrument control. Data processing was done using TargetLynx software and NAD metabolite was quantified by using external calibration curves.
