# Supplementary figures and images for: Effective treatment of mitochondrial myopathy by nicotinamide riboside, a vitamin B3
Source: EMBO Mol Med. 2014 Apr 7;6(6):721–31. doi: 10.1002/emmm.201403943 (PMC4203351; doi:10.1002/emmm.201403943)

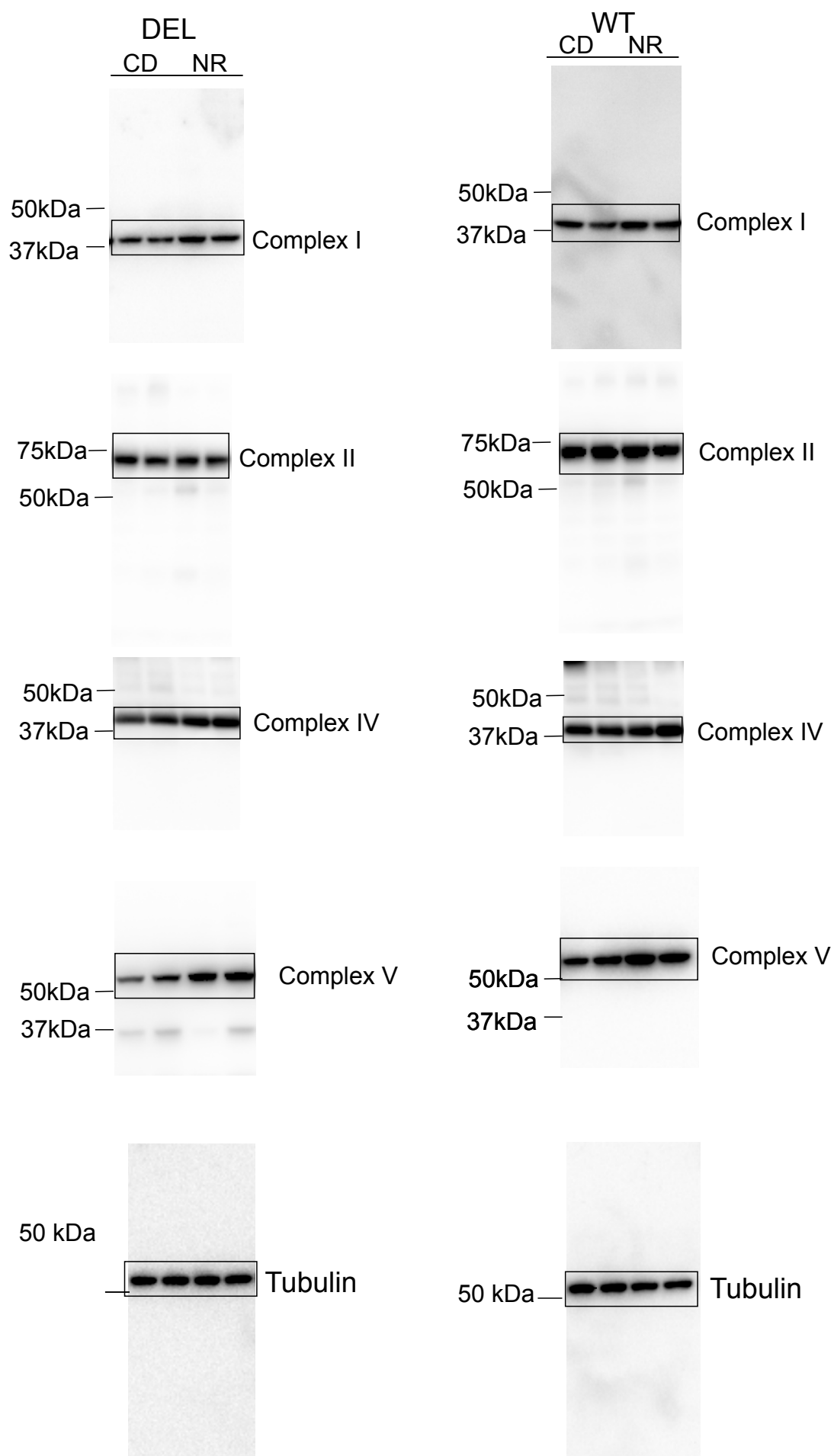

Fig 1D

Supplement: Supplementary file 6 — Source Data for Figure 1 [file emmm0006-0721-sd6.pdf]

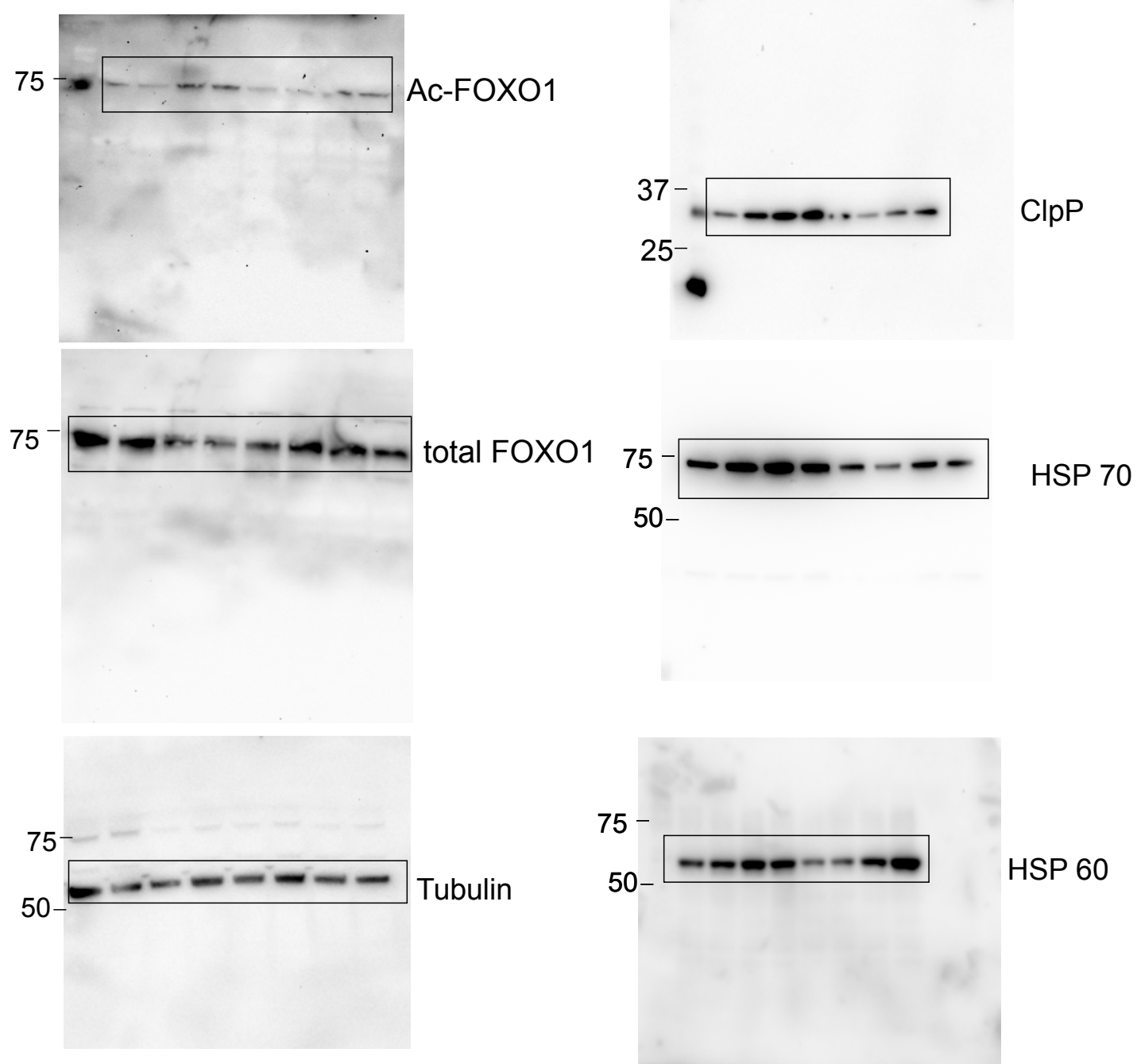

Fig 4A

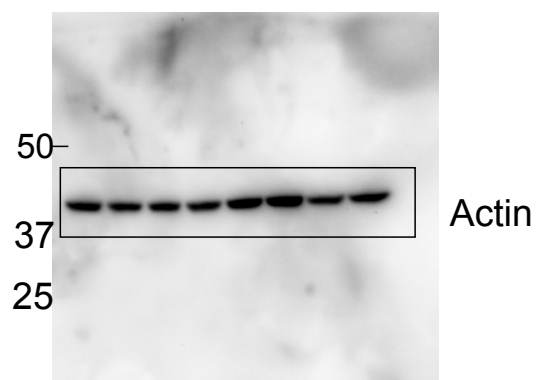

Fig 4G

Supplement: Supplementary file 7 — Source Data for Figure 4 [file emmm0006-0721-sd7.pdf]
